# SEC61G promotes breast cancer development and metastasis via modulating glycolysis and is transcriptionally regulated by E2F1

**DOI:** 10.1038/s41419-021-03797-3

**Published:** 2021-05-27

**Authors:** Jingjing Ma, Zhixian He, Hongwei Zhang, Wensheng zhang, Sheng Gao, Xiaojian Ni

**Affiliations:** 1 Department of Breast, Women’s Hospital of Nanjing Medical University, Nanjing Maternity and Child Health Care Hospital, Nanjing, 210004 China; 2 Department of General Surgery, Affiliated Hospital of Nantong University, Nantong, 226001 China; 3 Department of General Surgery, Zhongshan Hospital, Fudan University, Shanghai, 200032 China; 4 Cancer Center, ZhongShan Hospital, Fudan University, Shanghai, 200032 China; 5 State Key Laboratory of Genetic Engineering, MOE Engineering Research Center of Gene Technology, Key Laboratory of Reproduction Regulation of NPFPC and Collaborative Innovation Center for Genetics and Development, Fudan University, Shanghai, 200438 China

**Keywords:** Breast cancer, Prognostic markers

## Abstract

Breast cancer is the most common cancer in women and its incidence rates are rapidly increasing in China. Understanding the molecular mechanisms of breast cancer tumorigenesis enables the development of novel therapeutic strategies. SEC61G is a subunit of the endoplasmic reticulum translocon that plays critical roles in various tumors. We aimed to investigate the expression and function of SEC61G in breast cancer. By analyzing The Cancer Genome Atlas breast cancer cohort, we found that SEC61G was highly expressed in breast cancer and predicted poor prognosis of breast cancer patients. Overexpression of SEC61G and its prognostic role was also confirmed in the Nanjing Medical University (NMU) breast cancer cohort. Functionally, we demonstrated that knockdown of SEC61G suppressed breast cancer cell proliferation, migration, invasion, and promoted breast cancer cell apoptosis in vitro. Xenograft breast tumor model revealed that knockdown of SEC61G inhibited breast tumor development in vivo. Furthermore, we demonstrated that SEC61G positively regulated glycolysis in breast cancer cells. Mechanistically, we showed that transcription factor E2F1 directly bound to the promoter of SEC61G and regulated its expression in breast cancer cells. SEC61G overexpression antagonized the effect of E2F1 knockdown in regulating breast cancer cell proliferation, invasion, and apoptosis. Finally, we demonstrated that the E2F1/SEC61G axis regulated glycolysis and chemo-sensitivity of Herceptin in breast cancer cells. Taken together, these results of in vitro and in vivo studies demonstrate that SEC61G promotes breast cancer development and metastasis via modulating glycolysis and is transcriptionally regulated by E2F1, which might be utilized as a promising therapeutic target of breast cancer treatment.

## Introduction

Breast cancer is the most common cancer in women and accounts for one-fourth of all cancers^1,2^. The incidence rates of breast cancer are rapidly increasing in China, which contributes to 12% of all newly diagnosed breast cancer and 10% of all breast cancer deaths worldwide^3^. Breast cancer is a heterogeneous malignancy and can be classified into different molecular subtypes based on the expression of estrogen, progesterone, and HER2/neu receptor^4^. Treatment for breast cancer includes surgery, radiation therapy, chemotherapy, endocrine therapy, and targeted therapy^5^. In the past decade, substantial advances have been achieved in the treatment of breast cancer and the prognosis of breast cancer has been improved significantly^6^. However, the prognosis of patients with advanced breast cancer remains poor and resistance to systemic therapy needs to be addressed^7^. Hence, it is crucial to understand the molecular mechanisms of breast cancer development and metastasis and develop novel treatments to overcome drug resistance.

SEC61G, a subunit of the SEC61 translocon complex, plays an important role in protein folding, modification, translocation, and activation of the unfolded protein response^8^. Genome-wide analysis of gene expression in gastric cancer has identified that SEC61G was highly expressed in gastric cancer, which was further confirmed by immunohistochemical staining^9^. SEC61G was demonstrated to be a proto-oncogene in glioblastoma^10^. SEC61G was overexpressed in glioblastoma multiforme and knockdown of SEC61G suppressed tumor cell growth in response to endoplasmic reticulum stress^10^. A recent study further demonstrated that SEC61G could function as a novel prognostic biomarker to predict survival and response to therapies in glioblastoma patients^11^. Intriguingly, EGFR amplification in metaplastic breast carcinoma was found to be associated with the amplification of a discrete genomic region mapping to *SEG16G*^12^. Nevertheless, the expression pattern and function of SEC61G in breast cancer have not been fully understood.

In this study, we demonstrated that SEC61G was overexpressed in breast cancer and high expression of SEC61G predicted the poor outcome of breast cancer patients. SEC61G promoted breast cancer cell malignancy behavior both in vitro and in vivo, which functioned as an oncogene in breast cancer. We also identified that transcription factor E2F1 directly bound to the promoter of SEC61G and regulated its expression. Moreover, we revealed that the E2F1/SEC61G axis modulated glycolysis and Herceptin chemo-sensitivity in breast cancer cells. Taken together, our findings suggest that SEC61G could be utilized as a novel therapeutic target for breast cancer treatment.

## Results

### SEC61G is highly expressed in breast cancer and predicts poor prognosis of breast cancer patients

**Fig. 1:**
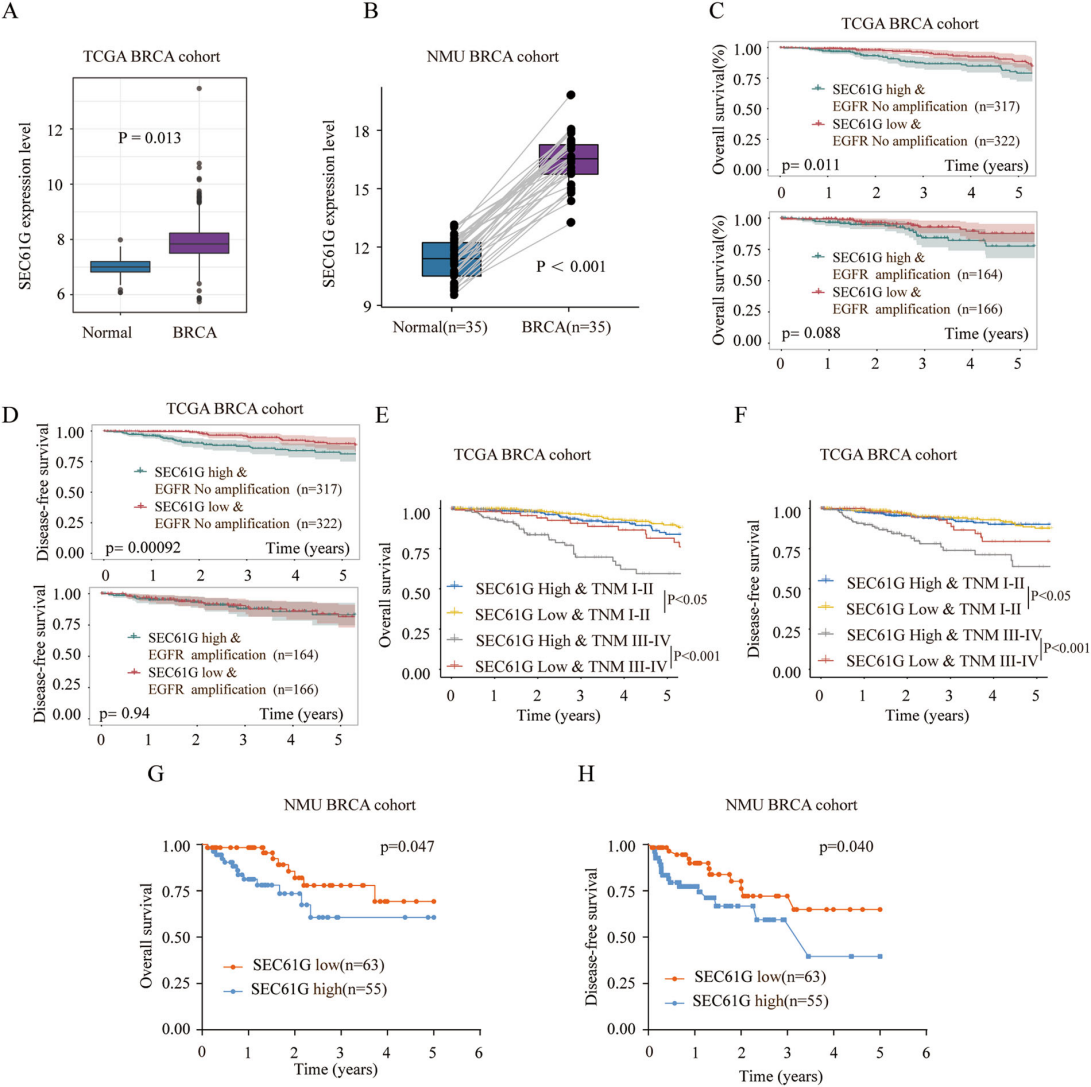
SEC61G is highly expressed in breast cancer and predicts poor prognosis of breast cancer patients in TCGA BRCA cohort and the NMU BRCA cohort. **A** The expression level of SEC61G in breast cancer tissues or normal control tissues in TCGA BRCA cohort was analyzed. *P* = 0.013. **B** The relative expression of SEC61G in breast cancer tissues or normal control tissues in the NMU BRCA cohort was analyzed by qRT-PCR (*n* = 35, *P* < 0.001). (**C**, **D**) Kaplan–Meier analysis of overall survival (OS) and disease-free survival (DFS) in breast cancer patients with high or low SEC61G expression in TCGA BRCA cohort, combined with EGFR amplification or not. **E**, **F** Kaplan–Meier analysis of the OS and DFS of breast cancer patients with high or low SEC61G expression at different TNM stages in TCGA BRCA cohort. **G**, **H** Kaplan–Meier analysis of the OS and DFS of breast cancer patients with high or low SEC61G expression in the NMU BRCA cohort.

To investigate the function of SEC61G in breast cancer, we first analyzed the expression of SEC61G in The Cancer Genome Atlas Breast Cancer (TCGA BRCA) cohort. SEC61G expression was significantly higher in breast cancer compared with that in normal tissues (Fig. 1A, *P* = 0.013). We also confirmed that breast cancer tissues had markedly higher expression of SEC61G in the NMU breast cancer (NMU BRCA) cohort (Fig. 1B, *n* = 35, *P* < 0.001). Prognosis analysis in the samples without EGFR amplification demonstrated that breast cancer patients with high expression of SEC61G had significantly worse overall survival (OS) and disease-free survival (DFS) than those patients with low expression of SEC61G in TCGA BRCA cohort (Fig. 1C, D). Furthermore, high SEC61G expression in breast cancer patients with tumor, nodes, and metastases (TNM) stage I–II or TNM III–IV also exhibited markedly lower rates of overall survival and disease-free survival compared with patients with low SEC61G expression (Fig. 1E, F). Consistently, the overall survival and disease-free survival rates of breast cancer patients with high SEC16G expression were remarkably lower than those in patients with low SEC16G expression in the NMU BRCA cohort (Fig. 1G, H).

**Fig. 2:**
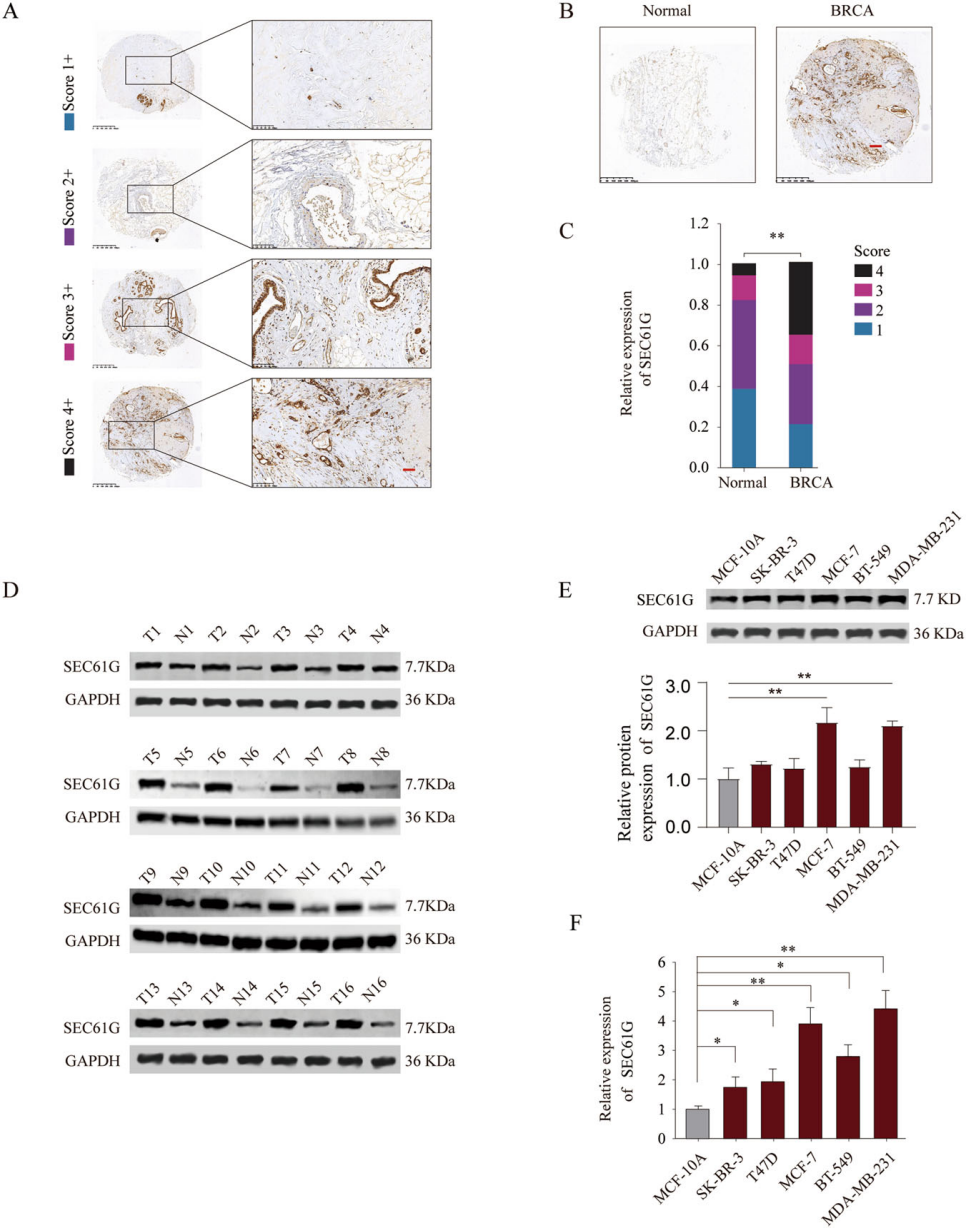
SEC61G expression is upregulated in breast cancer tissues and cell lines. **A** Representative SEC16G immunohistochemical (IHC) with different scores based on staining intensity in breast cancer tissue microarray. **B** Representative SEC61G IHC staining in breast cancer tissues and adjacent non-tumor normal tissues. **C** Comparison of IHC staining score distributions between breast cancer tissues and adjacent non-tumor normal tissues. **D** The protein expression of SEC61G in 16-paired breast cancer tissues (T) and adjacent normal tissues (N) was assessed by western blot. **E**, **F** The protein and mRNA expression of SEC61G in normal breast epithelial cell MCF-10A and breast cancer cell lines (SK-BR-3, T47D, MCF-7, BR-549, and MDA-MB-231) was analyzed by western blot or qRT-PCR. *P < 0.05, **P < 0.01.

Immunohistochemical staining of SEC61G was performed and the expression of SEC16G was scored based on the staining intensity in breast cancer tissues (Fig. 2A). The results demonstrated that breast cancer tissues had significantly higher expression of SEC61G in comparison with that in normal adjacent tissues (Fig. 2B, C). SEC61G protein in 16-paired breast cancer tissues and adjacent normal tissues was assessed by western blot. As shown in Fig. 2D, breast cancer tissues had much higher expression of SEC61G than that in normal tissues. To study the function of SEC61G in vitro, we examined the expression of SEC61G in different breast cancer cell lines and normal breast epithelial cell line MCF-10A. In comparison with MCF-10A, breast cancer cell lines including SK-BR-3, T47D, MCF-7, BT-549, and MDA-MB-231 had remarkably higher expression of SEC61G, both at protein and mRNA levels (Fig. 2E, F).

### Knockdown of SEC61G suppresses breast cancer cell proliferation, migration, invasion, and promotes breast cancer cell apoptosis in vitro

**Fig. 3:**
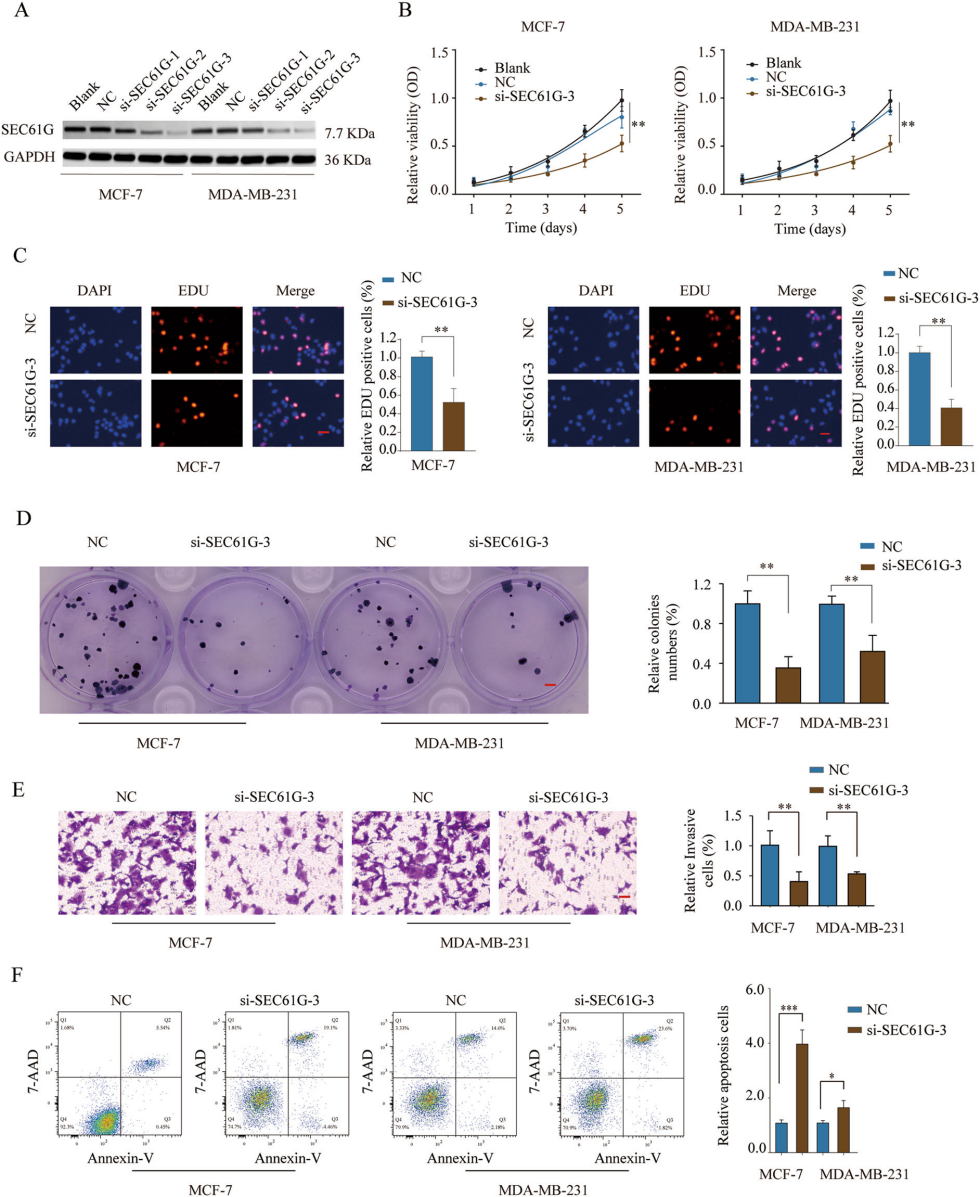
Knockdown of SEC61G suppresses breast cancer cell proliferation, migration, invasion, and promotes breast cancer cell apoptosis in vitro. **A** MCF-7 or MDA-MB-231 cells were left untreated (Blank), or transfected with negative control (NC) or siRNAs targeting SEC61G. The relative protein expression of SEC61G in MCF-7 or MDA-MB-231 cells was analyzed by western blot. **B**–**D** MCF-7 or MDA-MB-231 cells were left untreated or transfected with NC or si-SEC61G-3. **B** Cell proliferation was analyzed by CCK-8 assay at the indicated time points. **C** DNA synthesis was analyzed by the EDU incorporation assay. **D** Cell growth was assessed by colony formation assay. **E** Cell invasion was assessed by transwell assay. **F** Cell apoptosis was analyzed by Annexin V/7-AAD double staining. *P < 0.05, **P < 0.01, ***P < 0.001.

To explore the function of SEC61G in breast cancer, we utilized siRNAs targeting SEC61G to knock down the expression of SEC61G in MCF-7 and MDA-MB-231 cells, which had the highest expression level of SEC61G (Fig. 3A). The most potent si-SEC61G-3 was used for the subsequent experiments. Functional assays demonstrated that knockdown of SEC61G significantly suppressed cell growth of MCF-7 or MDA-MB-231 cells, showing a decreased cell proliferation, much-reduced DNA synthesis, and inhibited colony formation (Fig. 3B–D). The wound-healing assay revealed that knockdown of SEC61G suppressed breast cancer cell migration (Fig. S1A), while transwell assay demonstrated that silencing SEC61G inhibited breast cancer cell invasion (Fig. 3E). Cell apoptosis in breast cancer cells with SEC61G knockdown was analyzed by Annexin V/7-AAD staining and the results showed that knockdown of SEC61G significantly enhanced breast cancer cell apoptosis (Fig. 3F). Consistently, we demonstrated that knockdown of SEC61G decreased the expression of anti-apoptosis protein Bcl-2 while increased the expression of pro-apoptosis protein Bax, Bak, and cleaved-Caspase-3 in MCF-7 and MDA-MB-231 cells (Fig. Supplementary 1, B). We also explored the function of SEC61G via overexpressing SEC61G in MCF-7 or MDA-MB-231 cells (Fig. Supplementary 2A). Overexpression of SEC61G enhanced cell growth and invasion of MCF-7 or MDA-MB-231 cells in vitro (Fig. Supplementary 2B–D). In order to demonstrate the specificity of the knockdown and exclude the off-target effect of si-SEC61G, we also utilized si-SEC61G-2 to knock down the expression of SEC61G in MCF-7 and MDA-MB-231 cells (Fig. Supplementary 3). In vitro cellular function experiments were carried out and the results suggested that knockdown of SEC61G using si-SEC61G-2 also suppressed breast cancer cell proliferation, migration, and invasion, with less efficiency compared with that of si-SEC61G-3 (Fig. Supplementary 3).

### Knockdown of SEC61G inhibits breast cancer xenograft tumor development in vivo

**Fig. 4:**
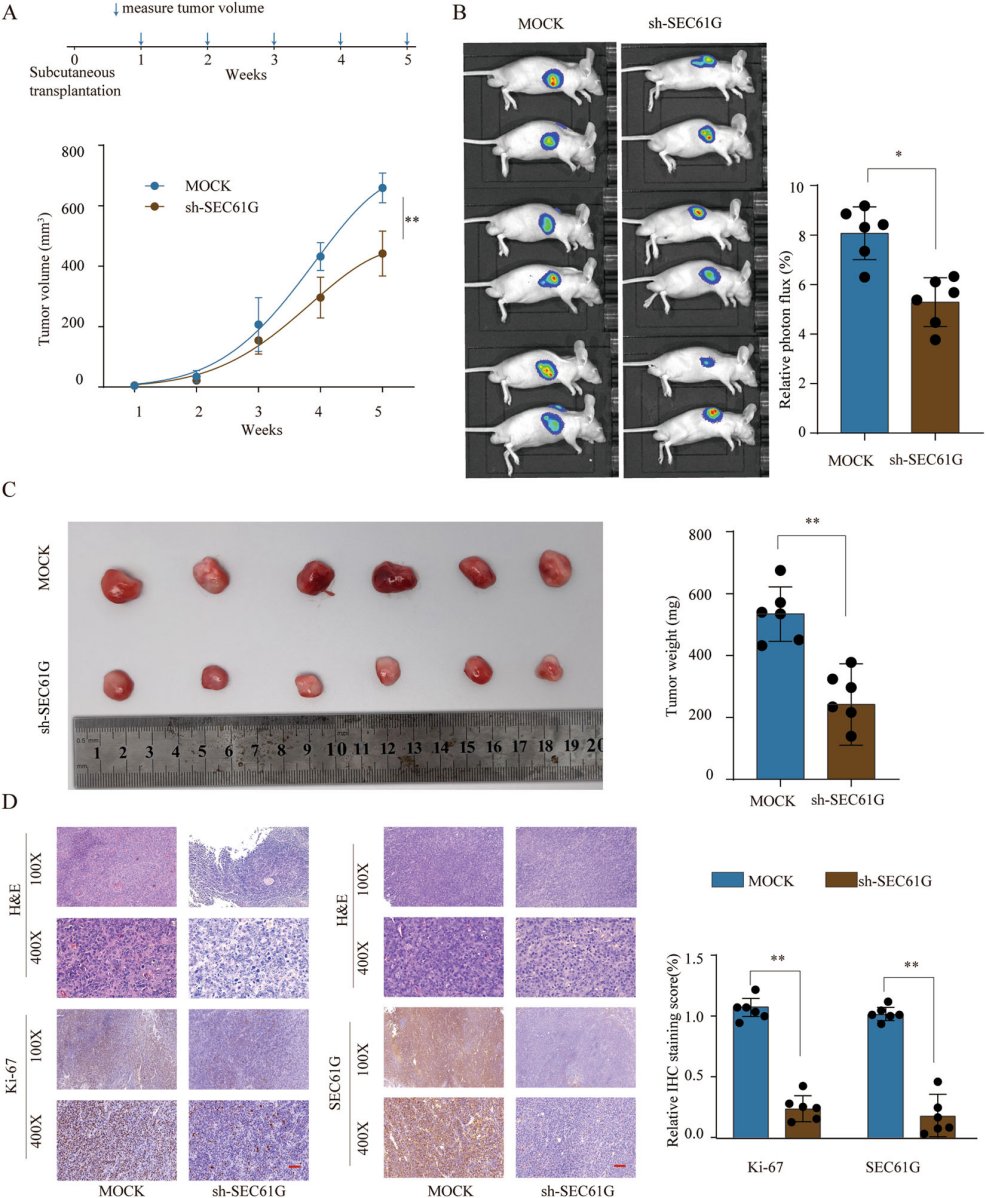
Knockdown of SEC61G inhibits breast cancer xenograft tumor development in vivo. Breast cancer MDA-MB-231 cells stably transfected with Mock or sh-SEC61G were implanted subcutaneously into nude mice to establish a xenograft tumor model and tumor growth was analyzed. **A** Growth curves of the tumor were examined based on tumor size measured every week (*n* = 6). **B** The relative photon flux of breast tumors in nude mice from the Mock or sh-SEC61G group was examined using a live imaging system to measure the luciferase signal. **C** Representative photographs of xenograft breast tumor tissues from the Mock and sh-SEC61G groups at week 5 were shown and tumor weights were analyzed. **D** Representative H&E staining and IHC staining images of SEC61G and Ki-67 of tumor sections from the Mock or sh-SEC61G group (left). Quantification of relative Ki-67 and SEC61G staining intensity in tumor sections from the Mock or sh-SEC61G group. *P < 0.05, **P < 0.01.

We further assessed the function of SEC61G in vivo using the xenograft tumor model. MDA-MB-231 cells stably transfected mock vector or sh-SEC61G were inoculated into nude mice and the xenograft tumor growth was monitored every week. Knockdown of SEC61G significantly suppressed tumor growth, showing a markedly smaller tumor size and decreased tumor weight (Fig. 4A–C). We also performed IHC staining of proliferation marker Ki-67 and SEC61G using xenograft tumor tissues (Fig. 4D). The results demonstrated that sh-SEC61G efficiently suppressed SEC61G expression in xenograft tumors and knockdown of SEC61G markedly inhibited Ki-67 expression (Fig. 4D). Thus, knockdown of SEC61G inhibited breast cancer xenograft tumor development in vivo.

### SEC61G positively regulates glycolysis in breast cancer cells

**Fig. 5:**
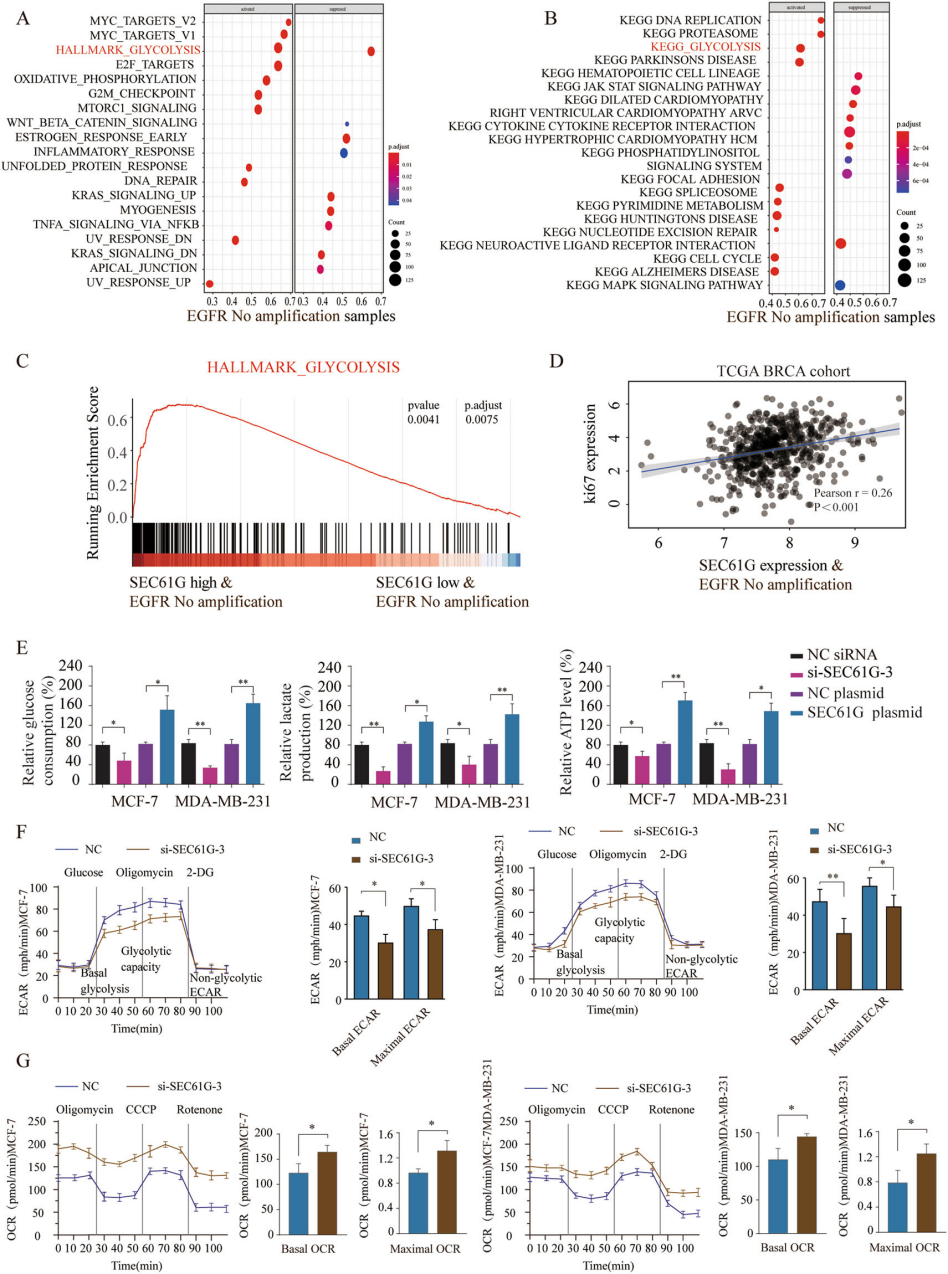
SEC61G positively regulates glycolysis in breast cancer cells. **A** Gene Ontology analysis (*P* < 0.001 FDR corrected) and **B** KEGG pathway enrichment analysis of the top 500 genes with high SEC61G expression in breast cancer cohort from TCGA database. **C** GSEA analysis of the relationship between SEC61G expression and glycolysis signaling. **D** Pearson analysis the correlation between SEC61G expression and Ki-67 expression in TCGA BRCA cohort. **E** MCF-7 or MDA-MB-231 cells were transfected with NC siRNA, si-SEC61G-3, NC plasmid, or SEC61G plasmid. The relative consumption of glucose, lactate production, and ATP levels in MCF7 or MDA-MB-231cells were analyzed. **F**, **G** MCF-7 or MDA-MB-231 cells were analyzed by Seahorse XP 96. Representative traces of the extracellular acidification rates (ECARs) and the oxygen consumption rates (OCRs) were shown respectively. The following compounds were injected into the assay microchambers as indicated: Glucose, oligomycin, carbonyl cyanide-4-(trifluoromethoxy)phenylhydrazone (CCCP), Rotenone, 2-deoxy glucose (2DG). Each data point is the mean ± SD of four technical replicates. *P < 0.05, **P < 0.01.

To further understand how SEC61G regulates breast cancer development, we performed Gene Ontology (GO) enrichment analysis and Kyoto Encyclopedia of Gene and Genomes (KEGG) signaling pathway analysis of the top 500 genes with the highest SEC61G correlation coefficient using the gene expression data of breast cancer cohort from TCGA database (Fig. 5A, B). The results indicated that glycolysis signaling was significantly enhanced in breast cancer patients with upregulated SEC61G expression (Fig. 5A, B). Besides, Gene Set Enrichment Analysis (GSEA) also demonstrated that high expression of SEC61G was positively correlated with glycolysis, indicating that SEC61G might regulate glycolysis in breast cancer development (Fig. 5C). Pearson correlation analysis suggested that SEC61G expression was positively associated with Ki-67 expression in TCGA BRCA cohort (Fig. 5D). To validate the hypothesis, MCF-7 or MDA-MB-231 cells were transfected with negative control siRNA or si-SEC61G-3, negative control plasmid, or SEC61G overexpression plasmid. We found that knockdown of SEC61G significantly inhibited glucose consumption, lactate production, and ATP levels in MCF-7 or MDA-MB-231 cells, while overexpression of SEC61G showed the opposite effect (Fig. 5E). Moreover, we used the Seahorse methodology to analyze glycolysis using the extracellular acidification rate (ECAR) and the mitochondrial oxidative phosphorylation activity using the oxygen consumption rate (OCR) in MCF-7 and MDA-MB-231 cells (Fig. 5F, G). Knockdown of SEC61G significantly suppressed glycolysis, showing decreased basal ECAR and maximal ECAR in breast cancer cells (Fig. 5F). In the contrast, knockdown of SEC61G markedly enhanced both basal and maximal OCR in MCF-7 and MDA-MB-231 cells (Fig. 5G).

### Transcription factor E2F1 directly binds to the promoter of SEC61G and regulates the expression of SEC61G in breast cancer cells

**Fig. 6:**
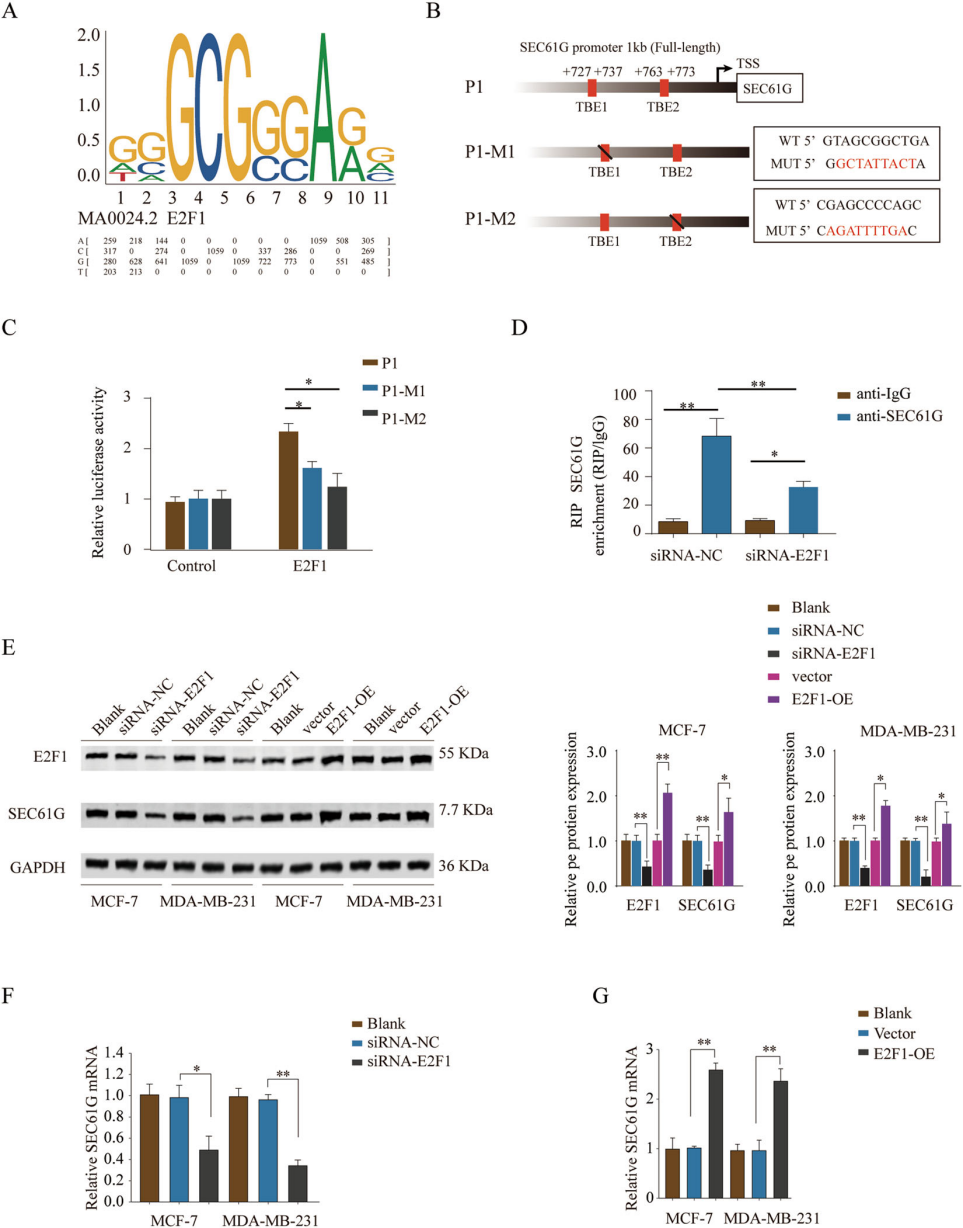
Transcription factor E2F1 directly binds to the promoter of SEC61G and regulates the expression of SEC61G in breast cancer cells. **A** The putative binding motif of E2F1 was predicted by using the JASPAR database (http://jaspar.generg.net) and Matrix profile. **B** The diagram showed the putative WT TEAD binding elements (TBE) located in the promoter region of the SEC61G gene. Luciferase reporter vectors containing WT TBE1/TBE2 (P1), mutated TBE1 (P1-M1), or mutated TBE2 (P1-M2) were constructed. **C** Luciferase reporter vectors containing WT or mutated TBE sequences were co-transfected with control or E2F1 overexpression vector into HEK293 cells. The relative luciferase activity was analyzed 48 h later. **D** HEK293 cells were transfected with siRNA-NC or siRNA-E2F1 and then RNA immunoprecipitation Chip (RIP) assay was performed using control IgG or SEC61G antibody. The SEC61G enrichment was analyzed by qPCR. **E**–**G** MCF-7 or MDA-MB-231 cells were transfected with siRNA-NC, siRNA-E2F1, empty vector, E2F1 overexpress vector (OE), or left untreated (Blank). **E** The protein expression of E2F1 and SEC61G was analyzed by western blot. GAPDH was used as a loading control. **F**, **G** The relative expression of SEC61G mRNA in MCF-7 or MDA-MB-231 cells was analyzed by qPCR. *P < 0.05, **P < 0.01.

To investigate how SEC61G was regulated in breast cancer, we performed bioinformatics analysis using the JASPAR database (http://jaspar.generg.net) and Matrix profile. We found that there were two putative TEAD binding elements (TBE) located in the promoter region of SEC61G gene (~1 kb) (Fig. 6A, B). Luciferase reporter vectors containing WT or mutated E2F1 binding sequences were constructed and co-transfected with control or E2F1 overexpression vector into HEK293 cells (Fig. 6C). The results showed that overexpression of E2F1 promoted luciferase activity while mutated either TBE1 or TBE2 resulted in diminished luciferase activity (Fig. 6C). RNA immunoprecipitation Chip (RIP) assay revealed that knockdown of E2F1 decreased SEC61G enrichment compared with that in the siRNA-NC control group (Fig. 6D). In addition, we found that knockdown of E2F1 significantly suppressed the protein and mRNA expression levels of SEC61G in MCF-7 or MDA-MB-231 cells, while overexpression of E2F1 enhanced SEC61G expression (Fig. 6E–G). Together, these findings suggested that E2F1 bound to the TBE1 and TBE2 in the SEC61G promoter region and regulated SEC61G expression.

### SEC61G antagonizes the effect of E2F1 knockdown in regulating breast cancer cell proliferation, invasion, and apoptosis

**Fig. 7:**
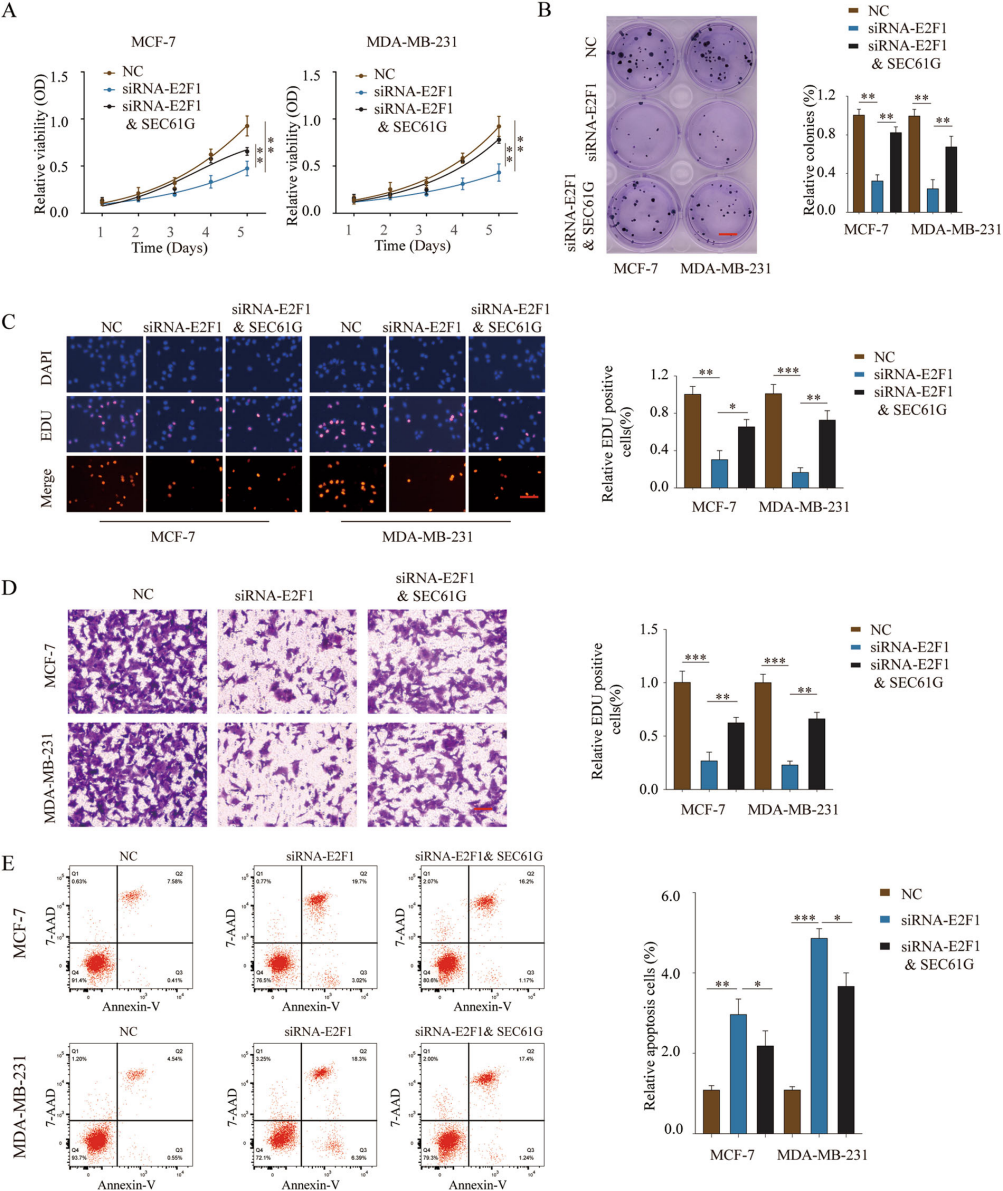
SEC61G antagonizes the effect of E2F1 knockdown in regulating breast cancer cell proliferation, invasion, and apoptosis. MCF-7 or MDA-MB-231 cells were transfected with negative control (NC), siRNA-E2F1, or siRNA-E2F1 & SEC61G overexpression plasmid. **A** Cell proliferation was analyzed by CCK-8 assay at the indicated time points. **B** DNA synthesis was analyzed by the EDU incorporation assay. **C** Cell growth was assessed by colony formation assay. **D** Cell invasion was assessed by transwell assay. **E** Cell apoptosis was analyzed by Annexin V/7-AAD double staining. *P < 0.05, **P < 0.01, ***P < 0.001.

To further study the functional relationship between SEC61G and E2F1, MCF7 or MDA-MB-231 cells were transfected with negative control, siRNA-E2F1, or siRNA-E2F1 & SEC61G overexpression plasmid. Functional assays demonstrated that knockdown of E2F1 markedly suppressed cell proliferation, EDU incorporation, and colony formation (Fig. 7A–C). However, the inhibition was partially blocked by SEC61G overexpression (Fig. 7A–C). Similarly, overexpression of SEC61G reversed the inhibition of cell invasion caused by E2F1 knockdown (Fig. 7D). In addition, knockdown of E2F1 promoted cell apoptosis of MCF-7 and MDA-MB-231 cells, while overexpression of SEC61G together with E2F1 knockdown antagonized the enhancement of cell apoptosis (Fig. 7E). Thus, SEC61G could antagonize the effect of E2F1 knockdown in regulating breast cancer cell proliferation, invasion, and apoptosis.

## Discussion

**Fig. 8:**
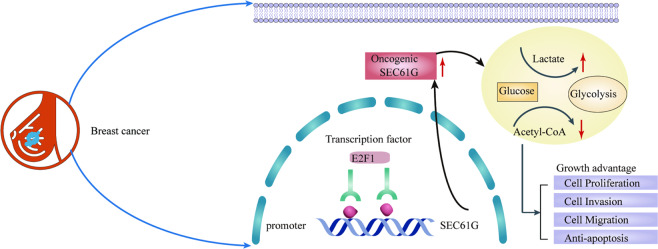
Schematic representation depicting the mechanisms that E2F1/SEC61G axis affects the progression of breast cancer. The diagram elucidates the functional axis of E2F1/SEC61G in regulating glycolysis and cell growth in breast cancer.

SEC61 complex is a heterotrimeric protein comprising three subunits, SEC61α, β, and γ^13^. Multiple studies have suggested that SEC61G is highly expressed in glioblastoma and inhibition of SEC61G could be a promising therapeutic strategy for glioblastoma^8,10,11^. Here we further demonstrate that SEC61G was highly expressed in breast cancer. The prognostic analysis suggested that high levels of SEC61G predicted the poor outcome of breast cancer patients. The oncogenic role of SEC61G in breast cancer was validated in both in vitro functional assays and in vivo xenograft tumor model. Mechanistically, transcription factor E2F1 positively regulated SEC61G expression via binding to the promoter region of SEC61G. High expression of E2F1 promoted glycolysis and provided growth advantage to breast cancer cells, as elucidated in Fig. 8. Thus, SEC61G could be used as a diagnostic biomarker and therapeutic target for breast cancer.

To understand the signaling pathway involved in SEC61G regulation, bioinformatics analysis was performed and glycolysis was highly associated with high expression of SEC61G. Glycolysis provides carbon skeletons, NADPH, and ATP for the survival and growth of breast cancer cells, even in the presence of oxygen^14^. Consistently, we showed that overexpression of SEC61G led to a high rate of glucose consumption, enhanced lactate production, and ATP levels while knockdown of SEC61G exhibited the opposite trend. Intriguingly, Pearson correlation analysis of TCGA BRCA cohort found that SEC61G expression was positively associated with proliferation marker Ki-67 expression, suggesting that upregulated SEC61G expression in breast cancer might be correlated with a high rate of cancer cell proliferation. However, how SEC61G modulates glycolysis remains unknown.

The regulation of SEC61G expression largely remains unknown. There was only one study showing that SEC61G was a direct target of miR-4677-ep in non-small cell lung cancer^15^. The most impressive finding in this study is that transcription factor E2F1 is directly bound to the promoter of SEC61G. Transcription factor E2F1 has a paradox role in tumor development, which can function as either oncogene or tumor suppressor^16^. Studies have shown that E2F1 overexpression links metastasis to chemo-resistance^17^. Here we revealed the oncogenic role of E2F1 in breast cancer via enhancing SEC61G expression. More importantly, we identified two putative E2F1 binding sites upstream of the SEC61G promoter and validated the binding via luciferase reporter assay. The interaction between E2F1 and SEC61G was further confirmed by the RIP assay. Future studies are needed to investigate the detailed signaling pathways involved in the E2F1/SEC61G axis regulating breast cancer development. Whether E2F1/SEC61G contributes to the metastasis of advanced breast cancer and targeting E2F1/SEC61G to improve drug-resistance in breast cancer treatment will be further studied.

Taken together, our findings demonstrate that SEC61G promotes breast cancer development and metastasis via modulating glycolysis and is transcriptionally regulated by E2F1. Thus, SEC61G could be utilized as a novel biomarker and therapeutic target for breast cancer treatment.

## Materials and methods

### Database analysis

The TCGA BRCA cohort data were downloaded from The Cancer Genome Atlas (TCGA, https://tcga-data.nci.nih.gov/tcga/). The expression profiles of SEC61G and its correlation with the prognosis of breast cancer patients were analyzed. The expression of SEC61G mRNAs was normalized to Log2 counts and then analyzed by BRB-array tools. Kaplan–Meier survival analysis and log-rank tests were performed for OS and DFS analysis.

### Cell culture and transfection

Human breast cancer cell lines (SK-BR-3, T47D, MCF-7, BT-549, and MDA-MB-231) and normal breast epithelial cell line MCF-10A were purchased from America Type Culture Collection (ATCC, Manassas, USA) and cultured with Dulbecco’s modified Eagle medium (DMEM) containing 10% fetal bovine serum (FBS, Gibco, USA) and 100 U/ml penicillin/streptomycin (ThermoFisher Scientific, USA). Cells were cultured in a humidified incubator with 5% CO_2_ at 37 °C. Transfection was done by using lipofectamine 3000 (Invitrogen, USA) following the manufacturer’s instructions. SiRNA targeting SEC61G, E2F1, and negative controls were purchased from GenePharma (Shanghai, China). ShRNA for stable knockdown of SEC61G or the negative control construct using a lentiviral system, and SEC61G overexpression plasmid was constructed and validated by GenePharma (Shanghai, China).

### Nanjing Medical University BRCA cohort

35-paired breast cancer specimens and adjacent normal control tissues were collected from breast cancer patients undergoing surgery at Nanjing Medical University during 2004 year to 2020 year (Nanjing Medical University BRCA cohort). All patients signed the informed consent and the study was approved by the Ethics Review Committee of Nanjing Medical University.

### Tissue microarray (TMA) construction and immunohistochemical (IHC) staining

The breast cancer tissue microarray (TMA) was constructed using the 35-paired breast cancer specimens and adjacent normal control tissues. Briefly, for each patient, a 1-mm diameter core of the tissue was punched from formalin-fixed paraffin-embedded tissues and arranged into the TMA blocks. IHC staining of SEC61G and Ki-67 was performed as previously described^18^. The expression of SEC61G in patient specimens was scored based on the staining intensity (range 1+ to 4+, negative to intensively strong). The antibodies used for IHC staining were purchased from Proteintech.

### Quantitative real-time PCR (qRT-PCR)

Total RNA from tissue specimens and cultured cells was purified using TRIzol reagent (Invitrogen, USA) and RNA concentration was measured. An equal amount of RNA (2 μg) was reverse transcribed into complementary DNA (cDNA) using Superscript Reverse Transcriptase (Applied Biosystems, USA). Quantitative real-time PCR (qRT-PCR) was performed on the ABI 7500 real-time PCR system (Applied Biosystems, USA) using the PowerUP SYBR Green Master Mix (Applied Biosystems, USA). The primer sequences of SEC61G and GAPHD were as following: SEC61G (amplicon size: 116 bp, Tm: 60 °C), 5′-AAAGGACTCCATTCGGCTGGTT-3′ (forward) and 5′-CAAAGAAGCCAATGAATCCC-3′ (reverse); GAPDH (amplicon size: 231 bp, Tm: 54 °C), 5′-GAGAAGGCTGGGGCTCATTT-3′ (forward) and 5′-AGTGATGGCATGGACTGTGG-3′ (reverse).

### Western blot analysis

Tissue specimens or cultured cells were lysed using 1× Cell lysis buffer (Cell signaling Technology, USA) containing protein inhibitor cocktail (Roche, Switzerland) and Protein concentration was measured using a BCA protein quantification kit (Pierce, USA). An equal amount of protein (20 μg) was separated by 10% SDS-PAGE and transferred onto a nitrocellulose membrane (Invitrogen, USA). The membranes were blocked with 5% non-fat milk at room temperature for one hour and then incubated with primary antibodies overnight at 4 °C. After washing with 1× TBST, the membranes were further incubated with Horseradish Peroxidase (HRP)-conjugated secondary antibodies at room temperature for 1 h. GAPDH was used as an internal loading control. The signals were detected using enhanced chemiluminescence (ECL) kit and the band intensity was calculated with the software ImageJ (NIH, Bethesda, USA). Primary antibodies used in the study were listed as the following: SEC61G (Proteintech, 11147-2-AP), Ki-67 (Proteintech, 27309-1-AP), E2F1 (Proteintech, 12171-1-AP), Bcl-2 (Proteintech, 12789-1-AP), Bak (ab32371), Bax (ab32503), Cleaved Caspase-3 (ab32042), and GAPDH (Proteintech, 60004-1-Ig).

### Cell growth assays

Cell growth was evaluated by CCK-8 assay, 5-Ethynyl-20-deoxyuridine (EdU) incorporation assay, and colony formation assay. Briefly, for CCK-8 assay, MCF-7 or MDA-MB-231 cells were seeded into 96-well plates (2000 cells/well) and cultured for indicated times. 10 μl CCK-8 reagent was added to cell culture 4 h before the measurement of absorbance at 450 nm. For EdU incorporation assay, MCF-7 or MDA-MB-231 cells were seeded into 24-well plates (50,000 cells/well) and cultured for 24 h, then the DNA synthesis rate was analyzed with an EdU staining Proliferation kit (Abcam, USA). Colony formation was done by seeding 500 cells into 6-well plates and measuring the cell colonies after 14 days. Cells were fixed with 4% paraformaldehyde (Sigma-Aldrich, USA) and stained with 0.5% crystal violet staining buffer (Sigma-Aldrich, USA).

### Wound-healing assay

Transfected MCF-7 or MDA-MB-231 were seeded into six-well plates and cultured until 90% confluence. An artificial wound was created using a sterile 200 μL tip. The floating cells were washed away using PBS and the remaining cells were cultured in serum-free medium for 48 h. The wound was recorded by an inverted microscope and cell migration was calculated.

### Transwell assay

Cell invasion was evaluated using a 24-well transwell chamber (Corning, USA). Briefly, 5 × 10^4^ transfected MCF-7 or MDA-MB-231 cells were suspended in 200 μL serum-free medium and added to the top chamber. Medium supplemented with 10% FBS (500 μL) was added to the bottom chamber. After incubation for 48 h, cells invading into the bottom chamber were fixed and stained with 0.5% crystal violet (Sigma-Aldrich, USA) and then counted.

### Cell apoptosis analysis

Transfected cells were cultured for the indicated time and then harvested for cell apoptosis analysis. Briefly, cells were washed with PBS and stained with Annexin V/7-AAD using an Apoptosis detection kit (BD Bioscience, USA). Apoptotic cells were detected by the FACSCalibur machine (BD Bioscience, USA).

### Luciferase reporter assay

Wild type (WT) of SEC61G promoter sequences containing E2F1 binding sites were amplified or mutated and then constructed into luciferase reporter vector (Promega, USA). HEK293 was transfected with the WT luciferase reporter vector (P1) or mutated luciferase reporter vector (P1-M1 or P1-M2), together with control vector or E2F1 overexpression plasmid. Relative luciferase activity was analyzed using a Dual-Glo Luciferase Reporter Assay System (Promega, USA) 48 h later.

### RNA immunoprecipitation assay

HEK293 cells transfected with siRNA-NC or siRNA-E2F1 were lysed with 1× cell lysis buffer (Cell Signaling Technology, USA) containing RNase inhibitor (ThermoFisher Scientific, USA) and protease inhibitor cocktail (Roche, Switzerland). Cell lysates were incubated with agarose beads conjugated IgG isotype control or anti-SEC61G antibody (Proteintech, 11147-2-AP) overnight at 4 °C. Then beads were washed with RNA binding buffer, followed by RNA extraction and qRT-PCR analysis of SEC61G enrichment.

### Glucose consumption, lactate, and ATP assays

Glucose consumption, lactate production, and ATP levels in MCF-7 or MDA-MB-231 cells transfected with SEC61G siRNA, SEC61G overexpression plasmid, or relative negative control were determined by using Glucose Uptake Colorimetric Assay kit, Lactate Assay kit, and ATP colorimetric assay kit (BioVision, USA) following the manufacturer’s instruction.

### Oxygen consumption rate (OCR) and extracellular acidification rate (ECAR)

MCF-7 or MDA-MB-231 cells were seeded into XF24 cell culture plates. The real-time oxygen consumption rate (OCR) and extracellular acidification rate (ECAR) were measured using the Seahorse Extracellular Flux Analyzer (Seahorse Biosciences, USA) to determine the oxidative phosphorylation and glycolysis. The analysis was conducted as previously described^19^.

### Xenograft tumor model

In vivo animal experiments were reviewed and approved by the Experimental Animal Ethics Committee of Nanjing Medical University. Male BABL/c nude mice (6-week old) were obtained from the Shanghai SLAC Animal Center (Shanghai, China) and randomly assigned into two groups (Mock group or sh-SEC61G group). 5 × 10^6^ of the MDA-MB-231 cells stably knockdown SEC61G or negative control cells were subcutaneously inoculated into the right flank of nude mice. Tumor size was measured by a vernier caliper every week and tumor volume was calculated (length × width^2^ × 1/2). In vivo tumorigenesis assays were also monitored with a bioluminescence imaging system (Caliper, USA). The mice were euthanized 5 weeks later and tumor weights were determined.

### Statistical analysis

Statistical analysis was performed using GraphPad Prism (V8.0, Prism, USA). Results were presented as mean ± standard deviation. The differences between groups were analyzed using Student’s *t-*test or one-way ANOVA. OS and DFS were analyzed using the Kaplan–Meier method and compared by the log-rank test. A *P* < 0.05 was considered statistically significant.

## Supplementary information

Supplementary Figure legends clean version

supplementary Figure S1

supplementary Figure S2

supplementary Figure S3

Supplementary Material 1-Ethical statement

Supplementary Material 2-WB

Supplementary Material 3-qPCR

Supplementary Material 4
