# Supplementary material for: SEC61G promotes breast cancer development and metastasis via modulating glycolysis and is transcriptionally regulated by E2F1
Source: Cell Death Dis. 2021 May 27;12(6):550. doi: 10.1038/s41419-021-03797-3 (PMC8155024; doi:10.1038/s41419-021-03797-3)
Supplement: Supplementary file 1 — Supplementary Figure legends clean version [file 41419_2021_3797_MOESM1_ESM.docx]

**Supplementary Figure S1. Knockdown of SEC61G suppresses breast cancer cell migration and promotes breast cell apoptosis.** MCF-7 or MDA-MB-231 cells were transfected with negative control (NC) or si-SEC61G-3. (A) The cell migration was analyzed by wound-healing assay. (B) The protein expression of Bcl-2, Bak, Bax, cleaved-Caspase-3 in MCF-7 or MDA-MB-231 cells was analyzed by western blot. GAPDH was used as a loading control. * P < 0.05, ** P < 0.01.

**Supplementary Figure S2. Overexpression of SEC61G promotes breast cancer cell growth, migration, and invasion in vitro.** (A) MCF-7 or MDA-MB-231 cells were transfected with control vector, SEC61G overexpression plasmid, or left untreated (Blank). The protein expression of SEC61G was analyzed by western blot. (B) Cell proliferation was analyzed by CCK-8 assay. (C) Cell growth was assessed by colony formation assay. (D) DNA synthesis was analyzed by the EDU incorporation assay. (E) Cell invasion was assessed by transwell assay. * P < 0.05, ** P < 0.01.

**Supplementary Figure S3. Knockdown of SEC61G using si-SEC61G-2 suppresses breast cancer cell proliferation, migration, and invasion *in vitro*.** MCF-7 or MDA-MB-231 cells were left untreated or transfected with NC or si-SEC61G-2. (A) Cell proliferation was analyzed by CCK-8 assay at the indicated time points. (B) DNA synthesis was analyzed by the EDU incorporation assay. (C) Cell growth was assessed by colony formation assay. (D) Cell invasion was assessed by transwell assay. * P < 0.05, ** P < 0.01.
