# Supplementary figures and images for: SEC61G promotes breast cancer development and metastasis via modulating glycolysis and is transcriptionally regulated by E2F1
Source: Cell Death Dis. 2021 May 27;12(6):550. doi: 10.1038/s41419-021-03797-3 (PMC8155024; doi:10.1038/s41419-021-03797-3)

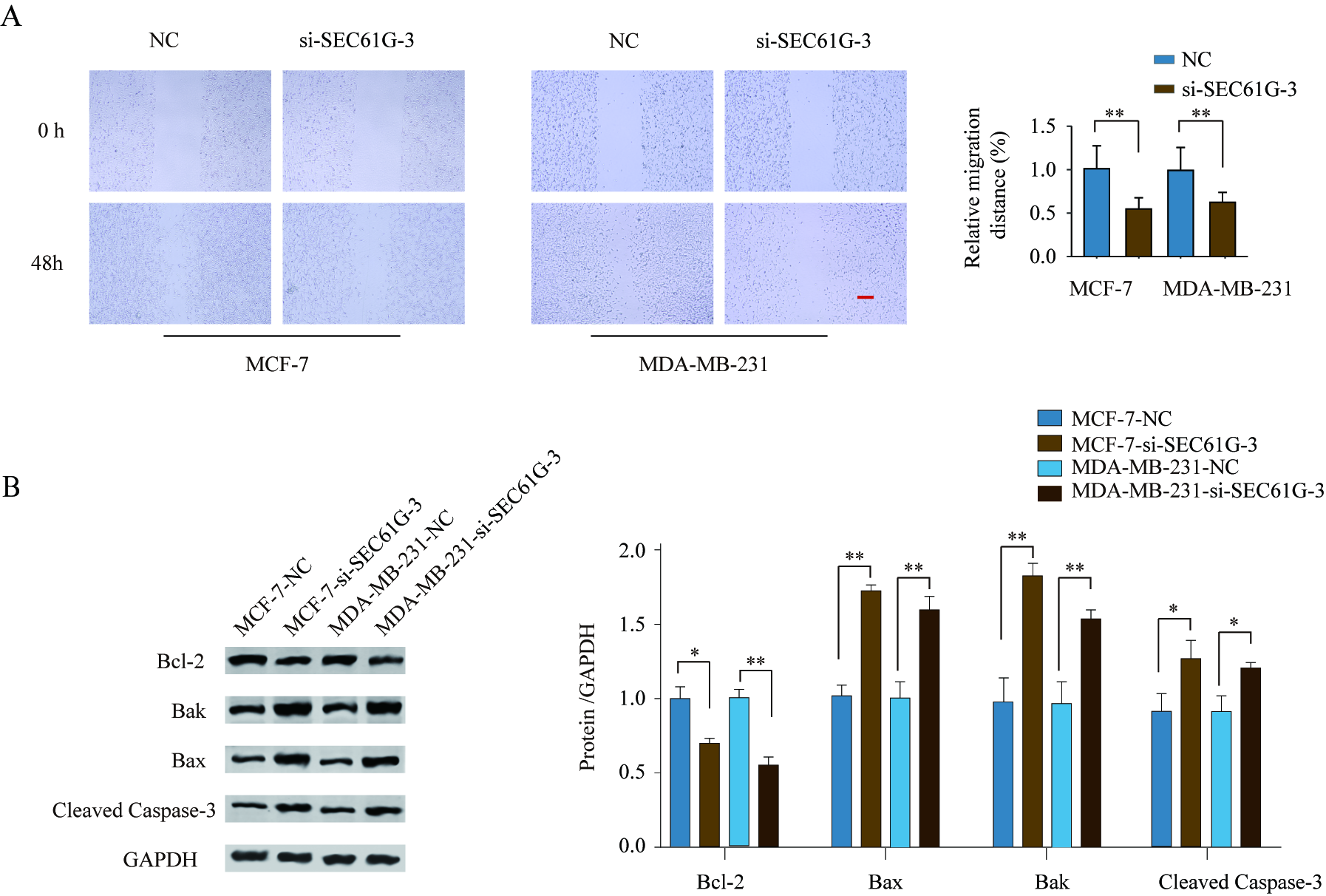

Supplement: Supplementary file 2 — supplementary Figure S1 [file 41419_2021_3797_MOESM2_ESM.tif]

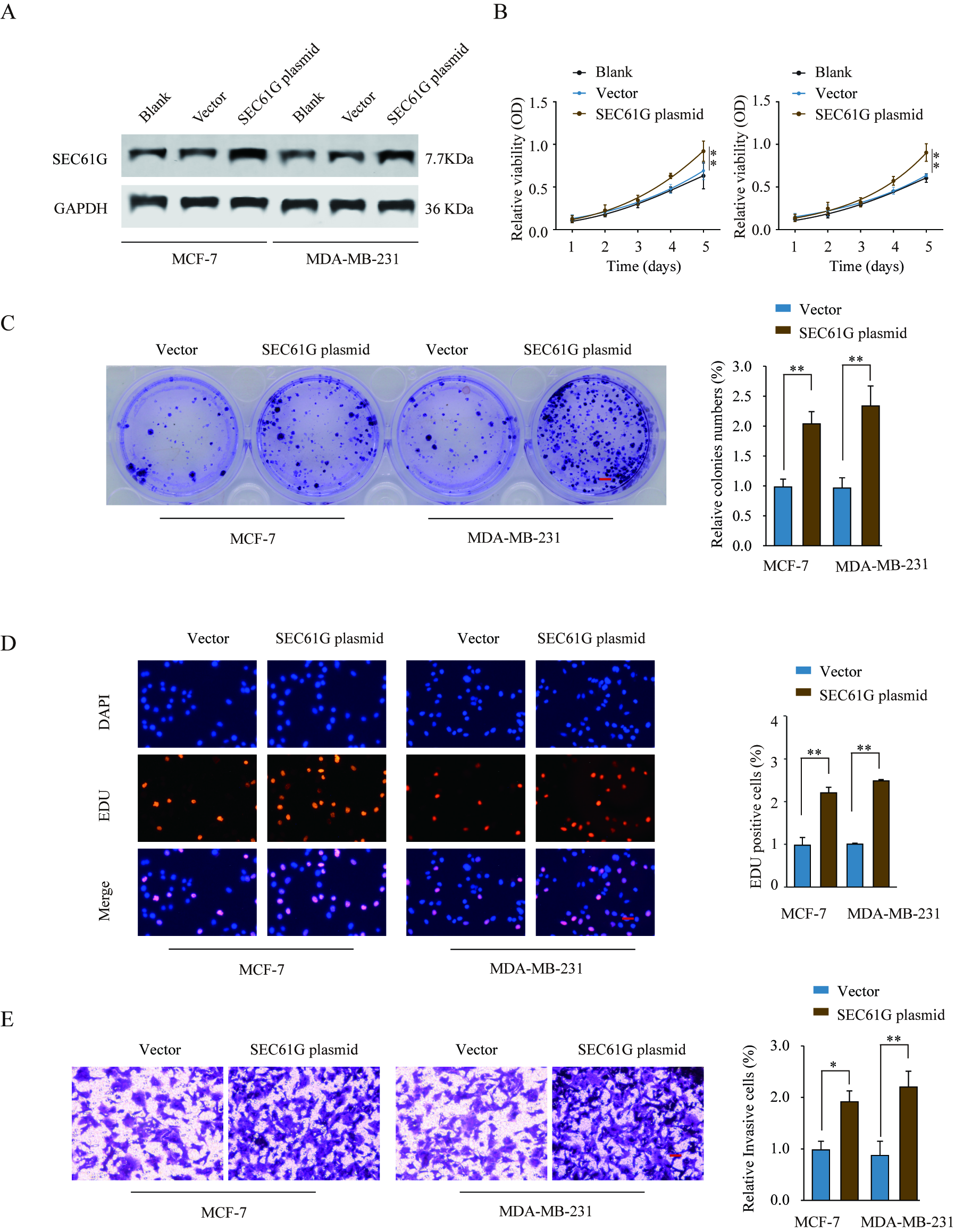

Supplement: Supplementary file 3 — supplementary Figure S2 [file 41419_2021_3797_MOESM3_ESM.tif]

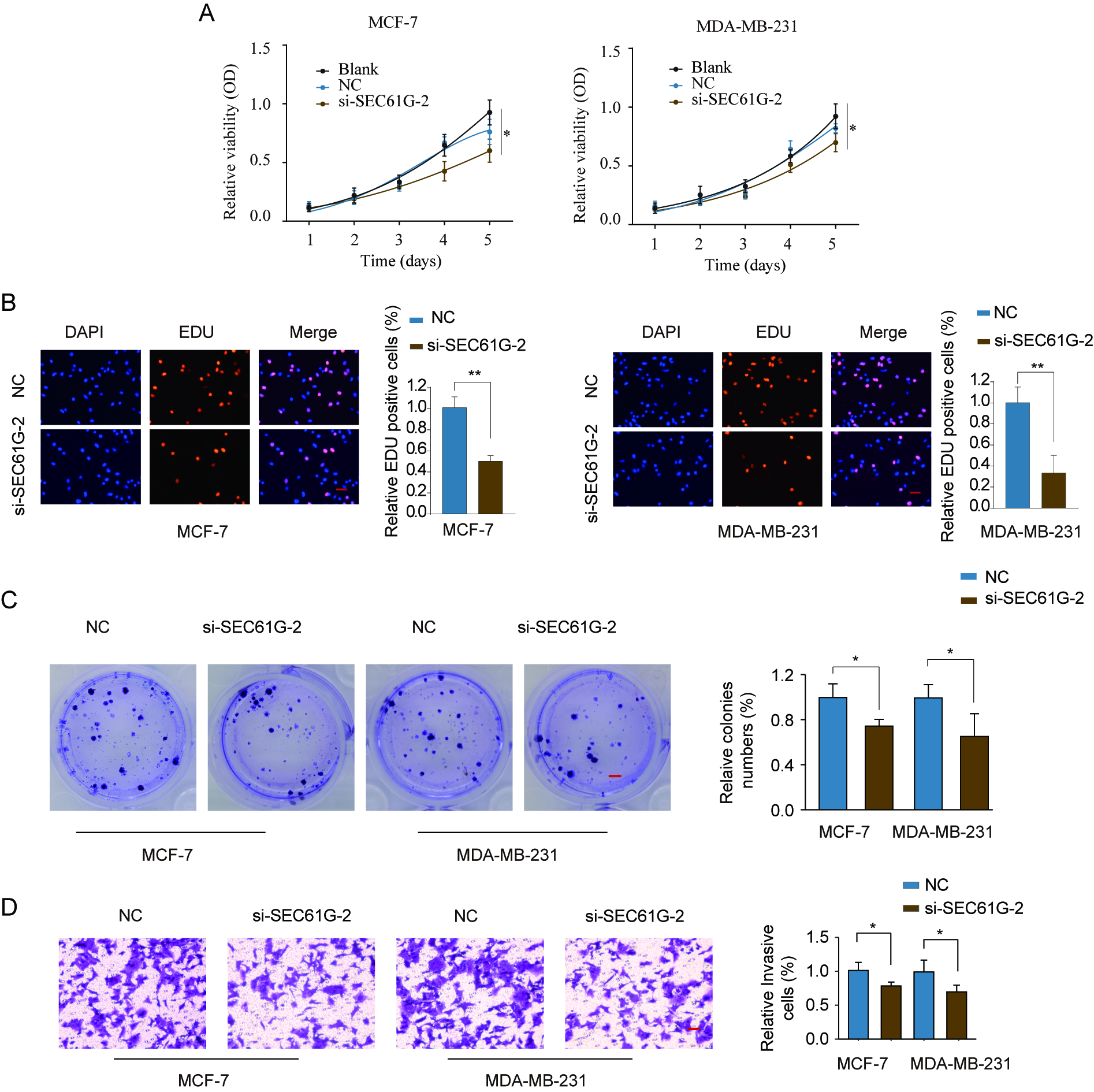

Supplement: Supplementary file 4 — supplementary Figure S3 [file 41419_2021_3797_MOESM4_ESM.tif]

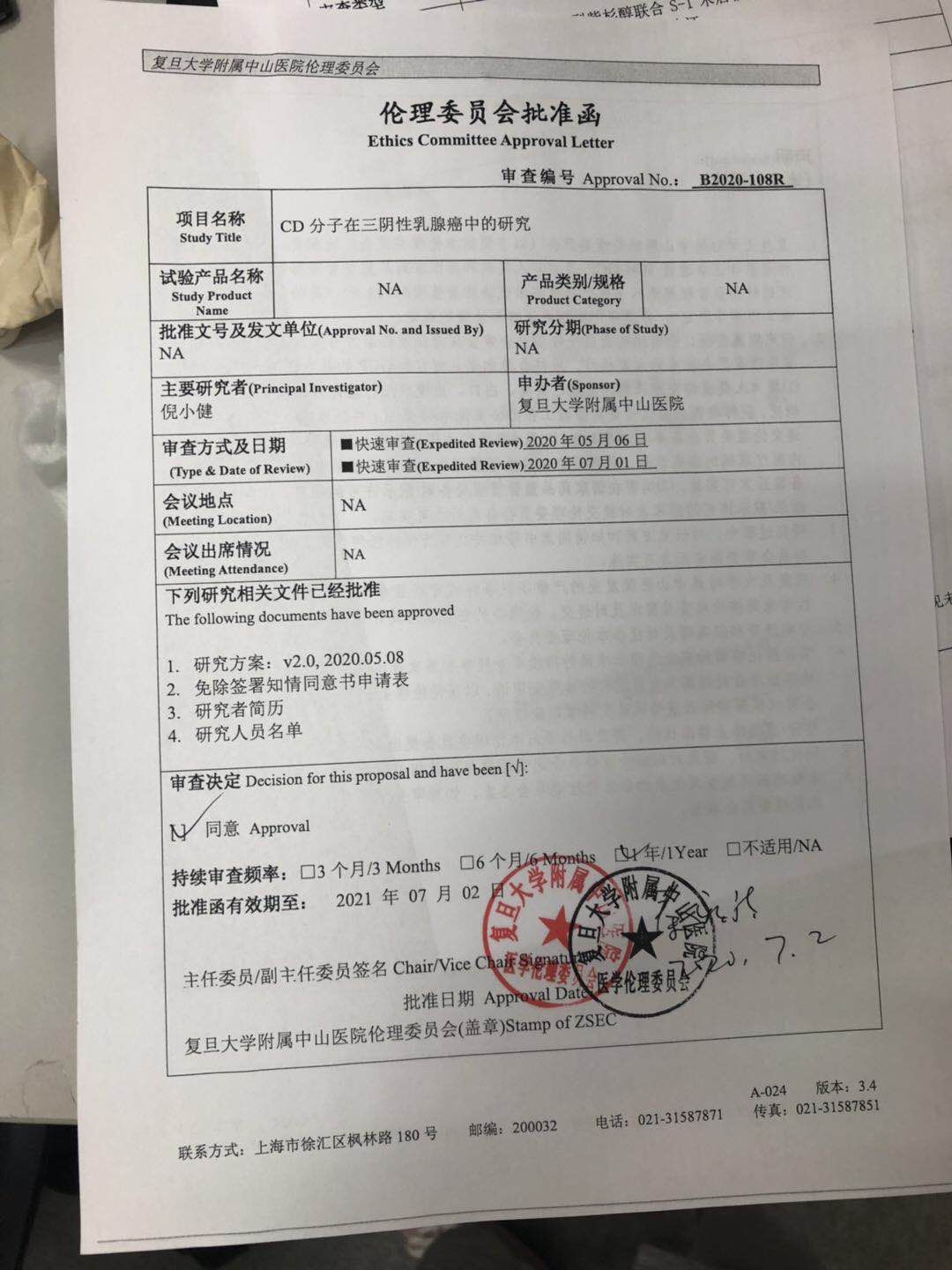

Supplement: Supplementary file 5 — Supplementary Material 1-Ethical statement [file 41419_2021_3797_MOESM5_ESM.jpg]

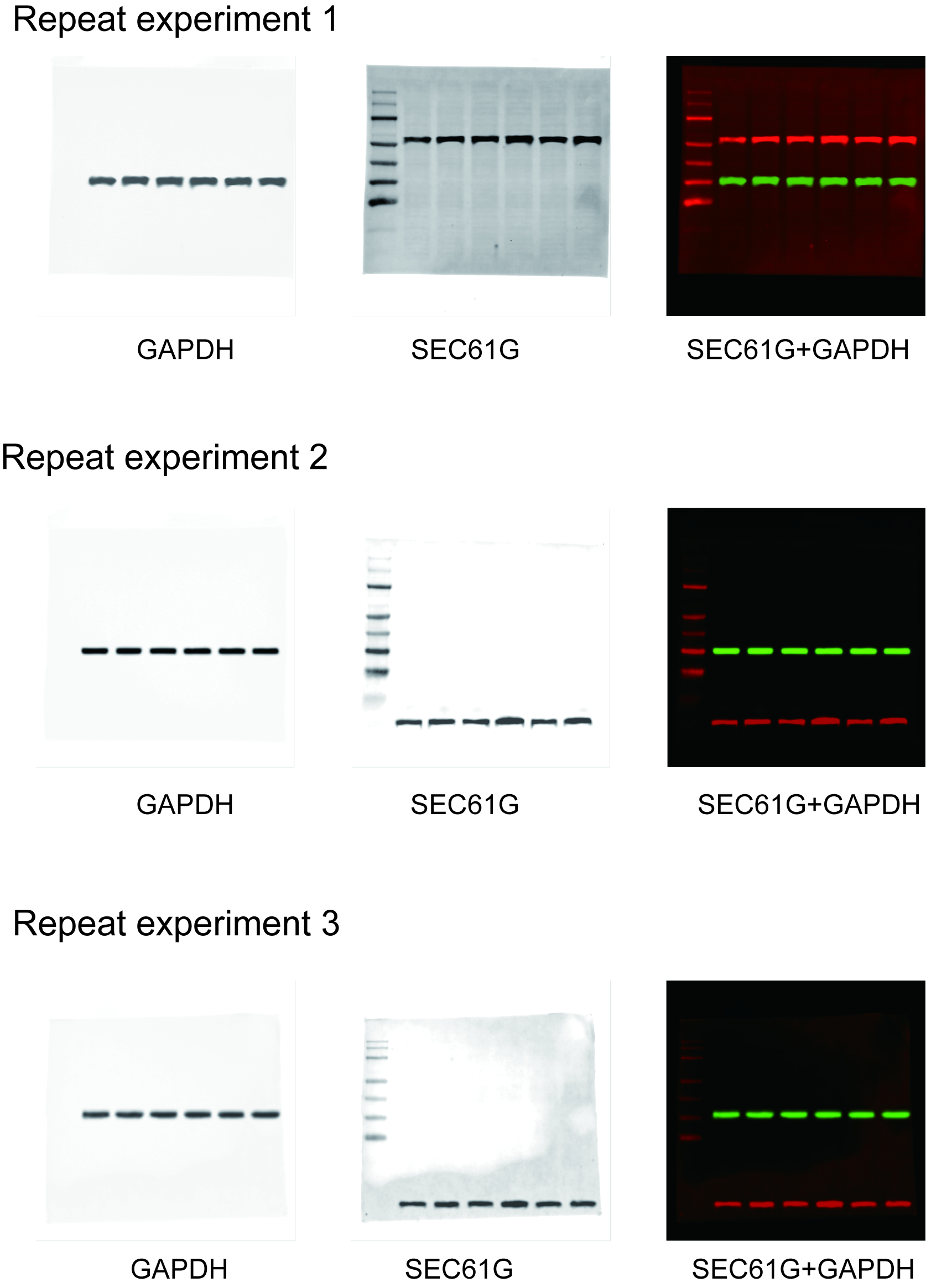

Supplement: Supplementary file 6 — Supplementary Material 2-WB [file 41419_2021_3797_MOESM6_ESM.tif]

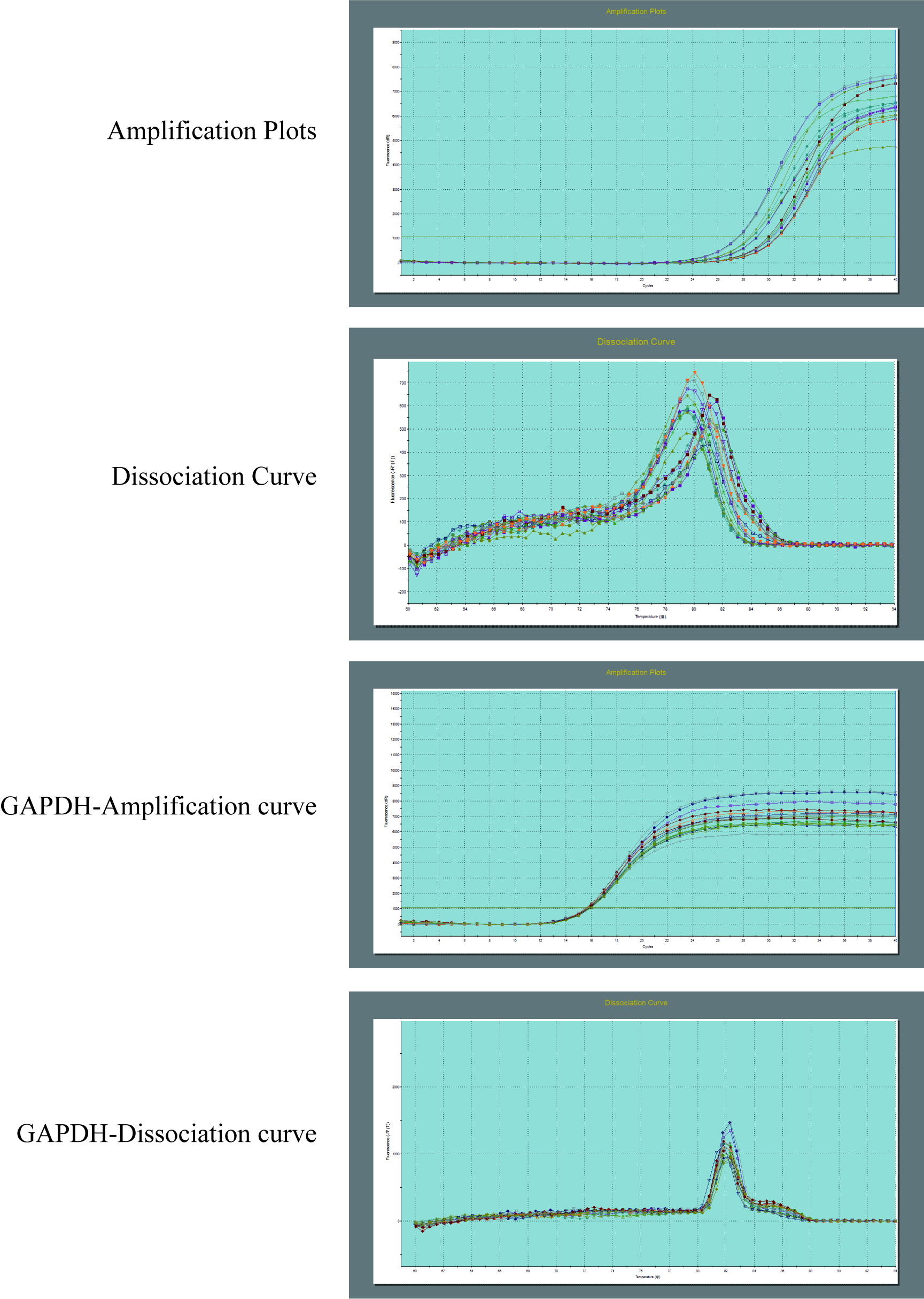

Supplement: Supplementary file 7 — Supplementary Material 3-qPCR [file 41419_2021_3797_MOESM7_ESM.tif]

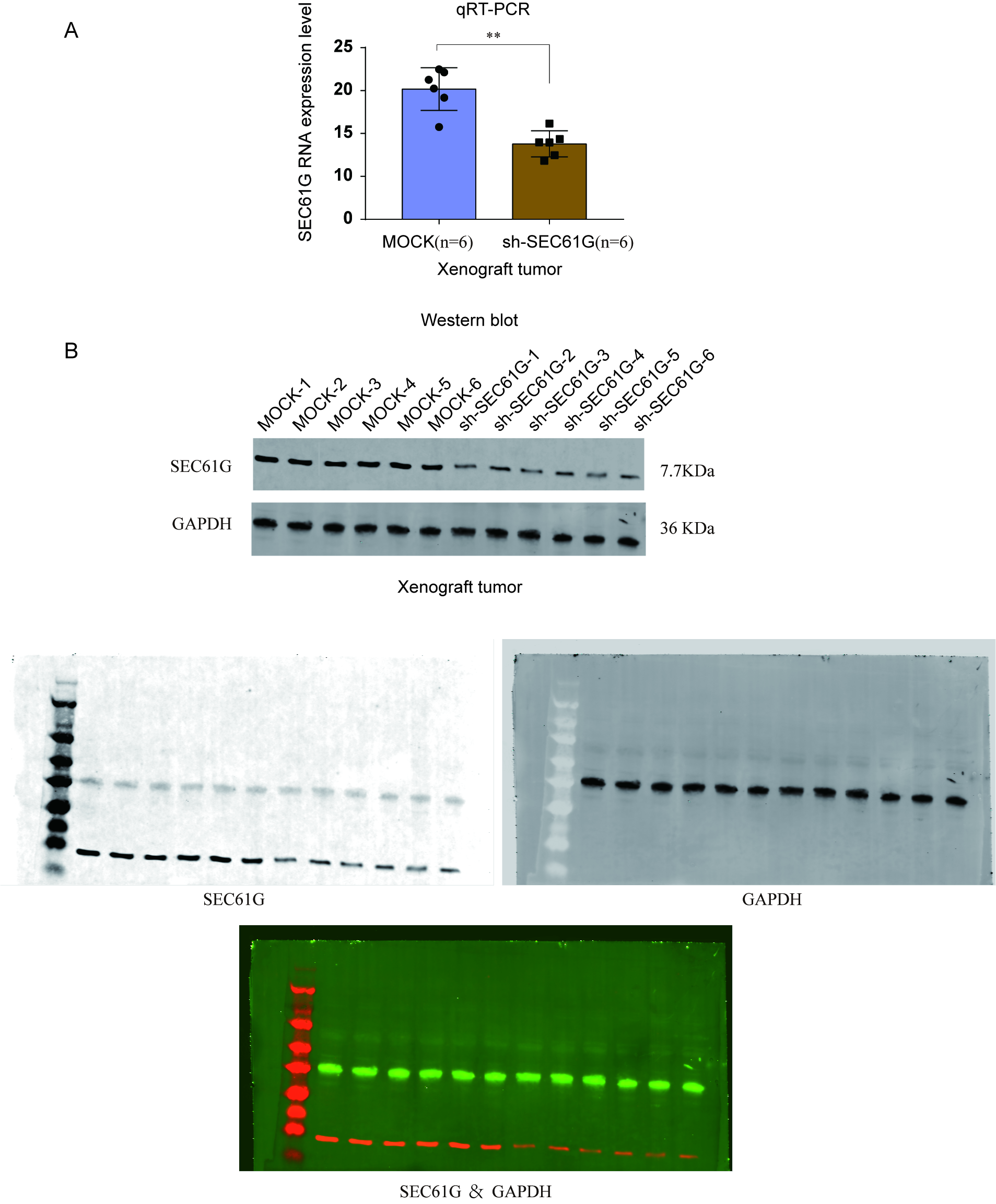

Supplement: Supplementary file 8 — Supplementary Material 4 [file 41419_2021_3797_MOESM8_ESM.tif]
